# Novel, Inexpensive, and Scalable Amyloid Fibril Formation Method

**DOI:** 10.3390/ma15051766

**Published:** 2022-02-26

**Authors:** Ethan Hessick, Milind Pawar, Reid Souchereau, Emma Schmitz, Pelagia-Irene Gouma

**Affiliations:** Advanced Ceramics Research Laboratory, Department of Materials Science and Engineering, The Ohio State University, Columbus, OH 43210, USA; pawar.38@buckeyemail.osu.edu (M.P.); souchereau.1@buckeyemail.osu.edu (R.S.); schmitz.151@buckeyemail.osu.edu (E.S.)

**Keywords:** amyloid, synthesis, inexpensive, melatonin biosensing

## Abstract

Wheat flour was used as a source of protein for the in vitro synthesis of Amyloid fibrils to develop a novel and inexpensive fabrication method. Amyloid fibrillation was confirmed by Thioflavin T Fluorescence, using confocal microscopy. A morphological study was carried out by transmission electron microscopy (TEM), which revealed the high aspect ratio of the amyloid fibrils formed via a novel process. An application of the amyloid fibers produced by the novel method is shown to be melatonin sensing. Tests showed that the amyloid samples had a measurable color variation dependent on the melatonin concentration. This newly derived process could prove to be a cost-effective tool for future nano-biomaterial applications in commercial and research settings.

## 1. Introduction

Amyloid fibrils were first observed by prominent 19th century German physician Rudolph Virchow in 1854 [1]. Ever since the model proposed by Pauling and Corey [2], the widely accepted structure for amyloid fibrils has been a cross-β core with β-strands embedded in a β-sheet and parallel to the fiber axis where β-sheets lie perpendicular to the major axis of fiber [3]. Amyloid fibrils can be obtained by the stable unfolding of functionally folded peptides or proteins [4]. The main steps involved in amyloid fibril formation, under suitable physio-chemical conditions, are protein modification from their natural state to unfolded intermediates, the modification of unfolded intermediates into parallel folded units, and the aggregation of these units into β strands that ultimately form the amyloid fibril. Amyloid fibril formation occurs as the transformation of an intermediate unfolded proteins’ structure into stacked β-strands of protein [4].

Recent studies have revealed that prefibrillar assemblies might contain toxic elements which are responsible for cell poisoning [5]. Diseases such as Alzheimer’s disease, Huntington’s disease, and Parkinson’s disease occur due to the accumulation of amyloid fibrils in tissues [6]. Since amyloid fibrils are insoluble and resistant to degradation, their formation causes the aforementioned diseases [7]. The analysis of the sequence and composition of amino acid revealed the specific proteins and peptides responsible for developing into dangerous amyloids, causing amyloid-based disorders [8]. However, there is an emerging field of functional amyloids utilizing the superior physical properties of amyloid fibrils, such as their strength, which is comparable to that of steel and silk fiber [9]. This suggests that amyloids can find applications as useful nanomaterials. The high Young’s moduli of amyloid fibrils is attributed to the dense network of hydrogen bonding, which leads to immense interactions between the backbones of polypeptides [10,11,12].

Studies focused on the behavior of amyloidogenic motif sequences provide better insight into the self-assembly mechanism of amyloid fibrils [13,14]. These studies provide motivation for designing amyloid-based nanomaterials for a wide variety of biomedical applications. Wetzel et. al. [15] summarized crucial similarities between amyloids, synthetic polymers, and plastics. Their four major summary points are:

1. Assembly properties of amyloid and its polymer subunits do not change under the influence of major chemical modifications.
2. Comparable isomorphism can be obtained from various monomeric units.
3. Condensed state formed via noncovalent interaction for both instances.
4. Under specific conditions, gel or liquid crystals can form.

The attractive features of amyloids include their high structural stability, nanoscale dimensions, and high stability at elevated temperatures. These versatile properties of amyloid fibrils provide ample opportunities for utilizing amyloids instead of DNA assemblies in nano-biotechnology. Amyloids have substantial potential to become functional materials. This potential is due to the fibrillar structure that can be used as a building block for nanomaterials, the slow reaction kinetics that allow for precise fabrication, tunability, and sensitivity to environmental factors, and general biocompatibility [16]. S. Bolisetty and R. Mezenga [17] incorporated amyloid fibrils within a carbon membrane for water purification applications. They demonstrated the role of amyloid fibrils in capturing heavy metal ions that can cause disease in the human body if left untreated. They also proposed a method to recover precious metals through an amyloid-based water purification system. S. Barrau et al. [18] integrated amyloid nanowires into organic solar cells to utilize amyloid fibrils as a template material for the electron donor-acceptor system in the developed solar cells, highlighting another application of amyloids as a promising nanomaterial for environmental engineering and energy applications.

Amyloid-based nanomaterials also have shown promise for medical and biotechnological uses. Wang et al. [19] created a nanostructure complex consisting of two antibodies connected by an amyloid nanorod. This dual antibody and amyloid structure allowed for the detection of unrelated cells or molecules or for functionalizing immune cells to better target cancer cells by binding one antibody to an immune cell and the other to a cancer cell. Castillo-Caceres et al. [20] developed a process for creating functional amyloids with enzymatic behavior. This is accomplished by manipulating the surface of amyloids to have regions resembling the active sites of enzymes. This process was utilized to create many enzymatic amyloids, including an amyloid that was able to assist in the hydrolysis of ATP, a vital reaction in cellular biology.

Recently, amyloids have also shown promise for biosensing applications. Díaz-Caballero, Navarro, and Ventura [21] created a system for designing amyloid-based biosensors. Their system functionalized β-sheets of an amyloid structure with biotin, then took advantage of the strong binding between biotin and streptavidin to attach streptavidin conjugates, such as enzymes, antibodies, or fluorescent biomolecules that could functionalize the complex for biosensing. They described a specific application of this system for glucose detection, by attaching horseradish peroxidase and glucose oxidase to their biotinylated amyloid fibrils, which produced a green color when glucose was present.

The use of amyloid-based materials in biotechnology, nanotechnology, and biosensing will continue to grow. Thus, it is important to address and research a major issue preventing their widespread use: due to the nature of the fabrication process of amyloids, expensive peptides or animal protein serums must be purchased and used. Purchasing pre-made amyloids is even more expensive. A common source of protein is bovine serum albumin (BSA). The wheat flour precursor used here offers a ten-fold decrease in the cost per gram of amyloid synthesis compared to BSA. This prompted an investigation into the synthesis of amyloid fibrils from an inexpensive source, namely wheat flour proteins. The complete characterization of the synthesized amyloid fibrils was performed and their analysis is presented. This process of amyloid fibril formation is scalable and will allow for the widespread use of amyloid-based nanomaterials in commercial or research applications.

To assess the functionality of amyloid fibrils produced from wheat flour, melatonin sensors were also produced and validated in this work for detecting low concentrations of melatonin. Research suggests that melatonin in high dosage may play a part in slowing or preventing Alzheimer’s disease due to possible interactions with amyloids involved in the process of neurodegeneration [22]. The interaction between amyloids and melatonin forms the basis of the novel biosensor. Therefore, in addition to proving the efficacy of the novel amyloid fabrication method, this work also demonstrates a novel and inexpensive melatonin biosensor system.

## 2. Materials and Methods

### 2.1. Materials and Chemicals

Gold Medal all-purpose flour was purchased from Kroger, Cincinnati OH, USA. Hydrochloric acid (HCl) and sodium dodecyl sulfate (SDS) were purchased from Thermo Fisher Scientific, Waltham MA, USA. Sodium chloride (NaCl), ethanol anhydrous, phosphate buffer saline (PBS) solution, Thioflavin T (ThT), Congo Red, and melatonin powder (>=98% purity) were all purchased from Sigma Aldrich, St. Louis MO, USA.

### 2.2. Amyloid fibrillation

Wheat flour contains different proteins that can be classified as albumins, globulins, gliadins, and glutenins [23]. Using the Osborne solubility rules [24], the separation of each protein was achieved. The only proteins to form amyloid fibrils under acidic conditions were the SDS-insoluble subunits of the glutenins. The aim of the separations was to isolate these SDS-insoluble glutenin subunits.

**Figure 1:**
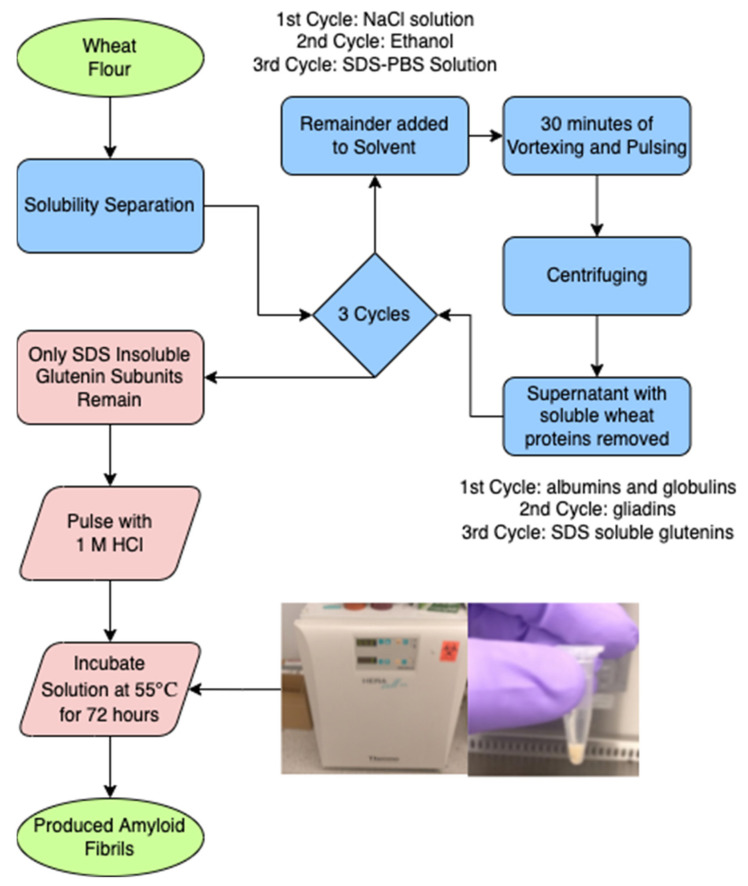
Simple flowchart of an overview of the amyloid synthesis process.

Each of the following separation steps used 0.4 mL of the described solution per 100 mg of wheat flour. The desired amount of wheat flour was weighed and added to an Eppendorf tube. Following this step, 2% (*w/v*) NaCl solution was prepared. Then, 0.4 mL of this solution was added to the Eppendorf tube for every 100 mg of initial wheat flour. The tube then alternated between undergoing between 5 min of pulsing and 5 min of vortex, for a total of 30 min after 3 repetitions. The tube was centrifuged for an additional 5 min. The proper mixing of the solution was ensured by vortex mixing. The supernatant of the albumins and globulins was removed from the mixture using a micropipette. Then, 70% (*v/v*) ethanol aqueous solution was prepared, and 0.4 mL was added for every 100 mg of initial wheat flour to the remaining pellet in the Eppendorf tube. The tube again alternated between undergoing 5 min of pulsing and 5 min of vortex, for a total of 30 min. Then, the tube was centrifuged for another 5 min. The supernatant of gliadins were removed from the mix using a micropipette. SDS phosphate-buffered solution was then prepared: 0.5% (*w/v*) of SDS was added to a 0.05 M PBS solution. This solution was heated using a hotplate with a magnetic stirrer until it was completely homogenized. Subsequently, 0.4 mL of the SDS-PBS solution was added (per 100 mg of initial wheat flour) to the pellet in the Eppendorf tube. The tube then alternated between undergoing 5 min of pulsing and 5 min of vortex, for a total of 30 min. Then, the tube was centrifuged for 5 min. Supernatant of SDS soluble glutenin subunits were removed from this mixture using a micropipette. After this entire separation process, the remaining pellet inside the Eppendorf tube only contained the SDS-insoluble glutenin subunits, which were shown to form amyloid fibrils. At this point, 1 M HCl at pH 1.6 was added to the remaining pellet. The solution was pulsated for 5 min until it became homogeneous and it was then placed inside the incubator at 55 °C for 72 h, when signs of fibrillation emerged. The overall process is summarized in Figure 1.

### 2.3. Characterization via Thioflavin T (ThT) Binding and Confocal Microscopy

Different methods are used for confirming the amyloid fibril formation. These include: (i) attaching Thioflavin T (ThT) and detection by fluorescence and (ii) morphological study by transmission electron microscopy (TEM), as was done in ref. [25].

Thioflavin T(ThT) is a benzothiazole dye with a high affinity for proteins which contain the β-sheet structure. ThT is widely accepted as the preferred stain to identify amyloid fibrils in a variety of different sample types, including both in vitro or ex vivo (see ref. [25]). Unbound ThT dye has a fluorescence excitation region from 385 to 450 nm. Upon binding to a region of protein with the β-sheet structure, ThT undergoes a characteristic spectral shift, increasing its fluorescence emission from 445 to 482 nm. This change in spectral shift is utilized for the bifurcation of bound ThT and unbound ThT. Ultimately, this spectral shift is used to identify amyloid fibrils where bound ThT is present [26].

A concentration of 3.14 mM Thioflavin T was made by dilution with distilled water. A volume ratio of 1:2 was used for the mixture of fibril solution to ThT solution. Then, 25 µL of the resulting solutions were taken from each sample and placed onto a glass slide. A cover slip was placed on top of the solution and edges were sealed with nail paint. The slides were then evaluated under an Olympus FV1000 Filter Confocal Microscope at Ohio State’s Campus Microscopy and Imaging Facility (CMIF), in Columbus OH, USA.

### 2.4. Characterization via Transmission Electron Microscopy (TEM)

TEM imaging was performed to confirm the presence of amyloid fibrils and study their morphology. The sample preparation method adopted in ref. [25] was also followed here to avoid artifacts in the micrographs obtained. TEM grids 200 copper mesh with formvar carbon coating were used, and the protein sample was negatively stained to produce contrast. First, 10 uL of amyloid sample was propped on the carbon-coated side of a TEM grid. Excessive solution was wicked off using a Kimwipe. Then, 10 uL of 2% uranyl acetate (UA) solution was dropped on a TEM grid containing the amyloid sample. After 3 min, excessive UA solution was wicked off using a Kimwipe and the sample was stored for a day in a desiccator. TEM analysis was performed after 24 hours using a FEI Tecnai G2 Spirit Twin TEM at 120 kV at Ohio State’s CMIF facility in Columbus OH, USA.

The TEM images were later analyzed using ImageJ to estimate the true mean fibril length and diameter from a randomly selected sample of 31 pictured fibrils. The line measure feature was used to collect the amyloid fibril length data. A 95% confidence interval was calculated using the normal distribution to estimate both parameters. The error for the confidence interval was calculated using the formula, $E=z_{\frac{a}{2}}\times \frac{\sigma }{\sqrt{n}}\;$, where *E* is the error, $z_{\frac{a}{2}}$ is the standardized normal distribution value corresponding to the selected confidence level (95%), σ is the standard deviation of the analyzed fibrils, and n is the number of analyzed fibrils. The fibrils’ mean aspect ratio was estimated by dividing the average length by the average diameter.

### 2.5. Melatonin Biosensing

In this work, amyloids were produced using a starting mass of 100 mg and the previously process described. After fibrillation, differing concentrations of melatonin were added to amyloid samples: 0 ng/mL (control), 2 ng/mL, 10 ng/mL, 50 ng/mL, and 100 ng/mL. Congo Red Dye was then added to the sample. Congo Red was selected to dye the amyloid for biosensing due to its established history of dying amyloids and orientation-based binding [27]. Thus, if there is an interaction between melatonin and the fabricated amyloid, the orientation or binding of the Congo Red may be affected, leading to an observable color change. The sensor input is the concentration of melatonin, and the sensor output is a change in the color of the dye.

Images of the samples were taken at two time intervals: 1 min after and 10 min after adding the Congo Red Dye. The images were subsequently processed using ImageJ to analyze each sample’s RGB values and grayscale values. ImageJ2 version 2.3.0 was used. ImageJ is an open-source tool available for download at https://imagej.nih.gov/ij/index.html (accessed on 13 September 2021). The rectangular select feature on ImageJ was used to select the pixels to be analyzed by the program. The measurements taken by the program were the mean, minimum, maximum, and the standard deviation of the grayscale value and RGB values on the selected region. Regions with color defects, such as bubbles, were excluded from the tested rectangle. These data were statistically analyzed and graphed, using Excel to calculate R^2^ values and MATLAB to plot mean color/grayscale value versus melatonin concentration. Version R2019b of MATLAB was used. Produced by Mathworks in Natick MA, USA. The data were again statistically analyzed and graphed, using Excel to calculate R^2^ values and MATLAB to plot the mean color/grayscale value versus the natural log of the melatonin concentration, after removing the data points for a melatonin concentration of 0. The first data set is a linear regression model, whilst the second is an exponential regression model.

## 3. Results

### 3.1. Characterization via Confocal Microscopy and ThT Binding

**Figure 2:**
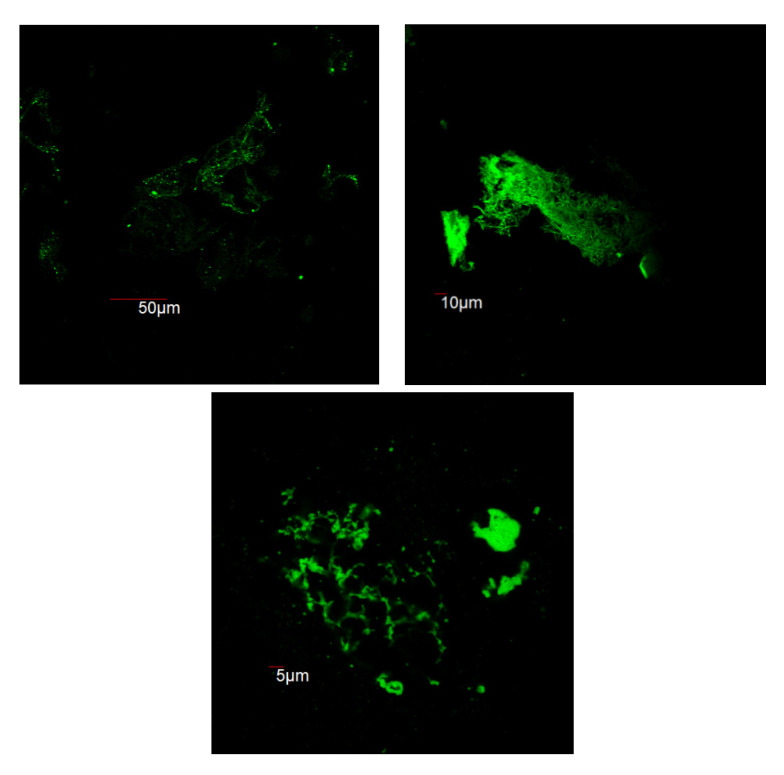
Confocal microscopy images of ThT-dyed amyloid fibrils taken at increasing magnification.

Due to the selective binding of the ThT dye to amyloids, fluorescence is observed to confirm the presence of amyloid fibrils. Figure 2 includes images taken by the confocal microscope for samples prepared at different magnifications.

The presence of bound ThT is proven by the confocal images taken in the enhanced ThT emission range. Bound ThT is an indicator of the folded structure of amyloid fibrils, so these images prove the successful synthesis of the desired material. Biancalana and Koide investigated the binding of ThT and amyloids on a molecular scale and found ThT to be an extraordinarily specific dye [26]. Confocal microscopy imaging does not allow for sufficiently high enough magnification to accurately measure the size of any of the amyloid fibrils but suggests that the size is in nanometers.

### 3.2. Characterization via TEM Imaging

**Figure 3:**
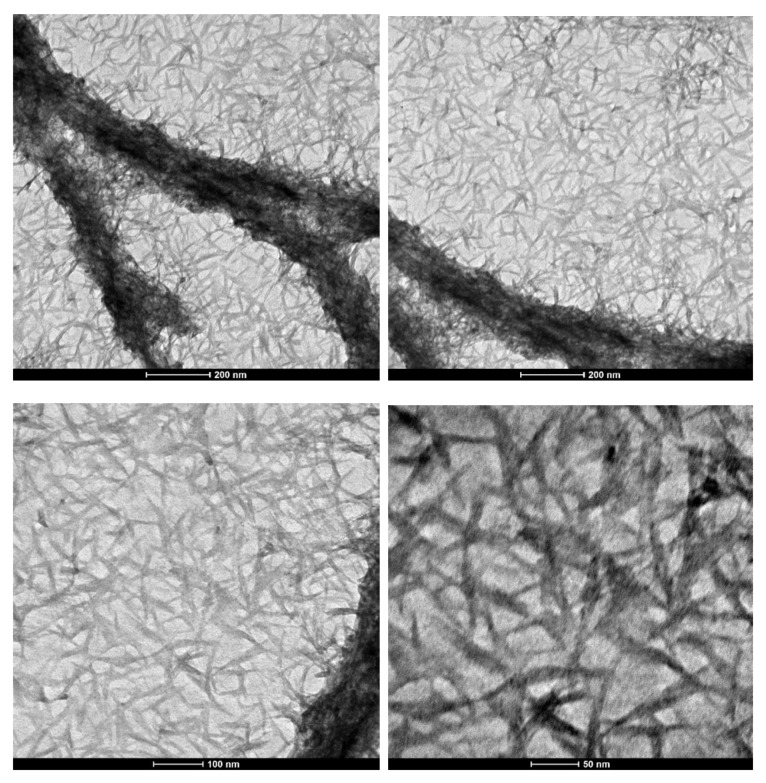
TEM images of amyloid fibrils at increasing magnifications.

Negative staining via uranyl acetate produced contrast and hence the amyloid fibrils appeared to be dark against a bright background. Figure 3 includes TEM images taken at different magnifications. It is evident that uniformly dispersed amyloid fibrils are present.

After sampling 31 of the pictured fibrils, the average fibril length was shown to be 86.87 ± 11.40 nm. The average fibril diameter was shown to be 8.57 ± 1.25 nm. Based on the average length and diameter calculations, an estimate of the amyloid fibril’s mean aspect ratio is 10.14 nm. The complete statistical breakdown of these amyloid fibrils is included in Table 1. Therefore, the materials obtained by the novel synthesis method are in the 10–100 nm range, which is considered to be the critical range for realizing novel material phenomena. Thus, these amyloids are an important system to study further.

FTIR spectroscopy could be performed in the future to investigate the functional groups on the surface of the amyloid fibrils. This would help develop a better understanding of the surface characteristics of the developed amyloids, which would benefit the further development of these amyloids into functional nano- or biomaterials.

### 3.3. Melatonin Biosensing

Melatonin is a hormone released by the pineal gland. It is known to be responsive to light and dark signals and plays a role in controlling and organizing circadian rhythms in the body. Important processes that rely on circadian rhythms include the sleep–wake cycle, actions of the immune system, hemostasis, and glucose regulation. Some research also suggests that melatonin has antioxidant properties and could be used to assist cancer treatments. Despite its proposed importance, relatively little has been proven regarding the role of melatonin in these processes [28]. Other preliminary research has shown that high doses of melatonin may prevent or slow down neurodegenerative diseases but this needs further study in clinical settings [22]. The high potential of melatonin as an efficient and effective medical treatment, combined with the fact that many individuals use melatonin tablets to combat episodes of insomnia, suggest that melatonin is an important biochemical analyte to measure and control. Biosensors in the literature typically measure the melatonin level in samples of human plasma, saliva, or urine. The prevalent methods for melatonin biosensing involve enzyme-linked immunosorbent assays (ELISA) or radioimmunoassay (RIA). Other methods include mass spectrometry and liquid chromatography. Current ELISA and RIA kits designed to measure melatonin levels in plasma and saliva have sensitivities as low as 1 pg/mL [29]. While these methods have great sensitivity and can measure extremely low concentrations of melatonin, they rely on expensive materials, such as highly specific antibodies.

**Figure 4:**
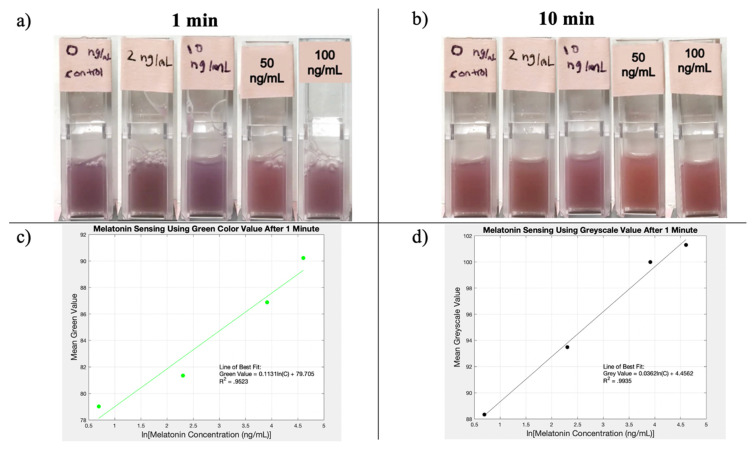
Results from the melatonin biosensing experiment including: (**a**) the melatonin sensing samples 1 min after addition of Congo Red Dye; (**b**) melatonin sensing samples 10 min after the addition of Congo Red Dye; (**c**) plot of mean green value vs. natural log of melatonin concentration based on the imaging of samples 1 min after addition of Congo Red Dye; (**d**) plot of mean grayscale value vs. natural log of melatonin concentration based on imaging of samples 1 min after the addition of Congo Red Dye. Graphs created on MATLAB.

Mean color values (red, green, and blue) were collected for the images taken of the melatonin sensing experiment after 1 min and after 10 min. These values were plotted on graph of mean color value vs. melatonin concentration for each color and time. The mean grayscale value was also plotted vs. melatonin concentration for both the 1 min samples and the 10 min samples. For each data set, the line of the best fit was plotted, and its R-squared value was calculated. This process was later repeated, using the color value vs. the natural log of the melatonin concentration, representing an exponential model. This model was found to better represent the relationship between the color change and the melatonin concentration. The exponential model had higher average R-squared values, which are the values reported in Table 2. The R-squared data for the linear regression model are included in Table A1 of Appendix A. The highest two R-squared values were for the mean green value after 1 minute and the mean grayscale value after 1 minute. For the exponential model, these R-squared values were 0.95 and 0.99, respectively. These two graphs, with their lines of best fit and r-squared values, are included in Figure 4. Additionally, Figure 4 displays the images of the melatonin sensing samples at both time intervals: 1 min and 10 min following the experiment.

The melatonin biosensing graphs show that the novel amyloid fabrication process has the potential to create viable biosensors. The two measurements highlighted in Figure 4 had relatively high R-squared values, indicating that the variance in the mean green value or mean grayscale value after 1 min was mostly explained by the dependent variable of the natural log of melatonin concentration.

Based on these data, it is reasonable to suggest that the described method was able to detect melatonin concentrations by analyzing the green color value or grayscale color value 1 minute after introducing the Congo Red Dye. However, there are some major limitations to this method that should be addressed.

First and foremost, the method needs a larger data set to truly prove its efficacy in measuring melatonin concentration. When more trials are performed, a more accurate relationship between the color values and the melatonin concentration will be found. More trials are necessary to determine which color value, such as grayscale or green color, is the most reliable detector before this method can be developed into a true, proven biosensor.

Then, the sensor sensitivity needs to be addressed. Based on the graphs in Figure 4, the sensor was unable to distinguish between the 0 and 2 ng/mL samples. The amyloid-based sensing only seemed to show a meaningful color change for the 10 ng/mL samples and above. Based on the collected data, the detection threshold is in the order of 10 ng/mL. Compared to other known melatonin biosensors in the literature, this is a relatively high value, which means that the sensor has low sensitivity. ELISA and RIA melatonin biosensors that are used to detect melatonin levels in plasma have sensitivities in the order of 1 pg/mL [29]. While this is a large difference, the amount of melatonin consumer by individuals to prevent jetlag is in the mg range, so consumers run the risk of melatonin overdose if they are not cautious. Furthermore, this high dosage of melatonin could be detected by the method described herein.

The cost-effectiveness, and potential for improvement of the sensing properties make this biosensing method a viable future tool. Taking advantage of a paired color detection method would help it become an over-the-counter detector. Many color-based sensors make use of phone apps to allow consumers to obtain accurate, highly sensitive reading based on a colorimetric biosensor, one such example being that from S. Zhang et al., where an app was designed in conjunction with the formaldehyde biosensor for the at-home detection of formaldehyde [30].

Addressing the time-dependency of the sensor’s output signal, the R^2^ values for each color value and the grayscale value were observed to be higher after 1 min than after 10 min. These data can be seen in Table 2. These data suggest that this biosensing method is highly time sensitive. Since only two times were tested, the optimal detection time interval still needs to be determined. A thorough, real-time time-based experiment would be very useful in determining the precise moment that the biosensors’ color change is most closely related to the melatonin concentration. More data on the time-dependency of this biosensing method could possibly help improve the sensitivity of the biosensing method and would also provide vital data for the future commercialization of this biosensing method.

## 4. Discussion

This study was carried out to explore a new synthesis method for amyloid fibrils. The scalability of the synthesis method was also an important and desirable factor. The scalable nature of the presented method allows for the industrial, commercial, and research applications of amyloid fibrils. Wheat flour was successfully used as a source protein for their synthesis. This was confirmed through characterization with confocal microscopy and TEM imaging. TEM analysis revealed the presence of amyloid fibrils with a relatively high aspect ratio. Amyloids have high research and commercial potential in many disciplines and applications. It was shown here that the produced amyloid fibers were capable of melatonin sensing. The presented synthesis method is expected to be applied to future nano biomaterial research and development.

The melatonin biosensing properties of the produced amyloid fibrils could be further researched to create a more sensitive melatonin biosensor. Further research into the interaction between Congo Red Dye, melatonin, and the produced amyloid fibrils would yield a better understanding of the sensing mechanism that could guide insights for improving its sensitivity or creating amyloid sensors for other important biomolecules or chemicals. If some of these questions are answered, the melatonin biosensing mechanism described herein has potential to be used commercially or for research purposes. The system could also be translated into other biomolecules or chemicals of interest that have amyloid interactions similar to amyloid–melatonin interactions.

Other applications of the cost-efficient amyloids described here should be tested. Current applications of amyloids, such as water-purification [17], targeting cancer cells [19], and producing enzyme-like behavior [20], to name a few, could be made more applicable and real-time by decreasing the costs and studying the structure of the produced amyloids at a molecular level. Research focusing on manipulating the molecular structure of the produced amyloids to assist with the binding of specific biomolecules or functional groups would achieve this goal.

## Figures and Tables

**Table 1:** Fibril measurement statistics based on a random sample of 31 of the amyloid fibrils from the TEM images. Data were collected using ImageJ software.

Amyloid Fibril Statistics (nm)					
Parameter	Average	Error	Standard Deviation	95% Confidence Interval	
				Lower Limit	Upper Limit
Length	86.87	11.40	32.37	75.48	98.27
Diameter	8.57	1.25	3.54	7.32	9.82

**Table 2:** R^2^ values for the exponential regression models for mean color values based on the natural log of melatonin concentrations. Sorted by the measured color and the time at which the measurements were taken after the Congo Red Dye was added. Values were calculated using Excel.

R^2^ Values for Color Value vs. ln(Melatonin Concentration)				
Time (Minutes)	Measured Color			
	Red	Green	Blue	Grayscale
1	0.78	0.95	0.62	0.99
10	0.56	0.36	0.01	0.64

## Data Availability

The raw data required to reproduce this study are available from M.P. upon reasonable request. The processed data required to reproduce this study are available from H.E. upon reasonable request.

## Appendix A

Table A1 includes the linear regression data for mean color values versus melatonin concentration.

**Table A1:** R^2^ Values for the linear regression models for mean color values based on the melatonin concentrations. Sorted by the measured color and the time at which the measurements were taken following the addition of the Congo Red Dye. Values calculated using Excel.

R^2^ Values for Color Value vs. Melatonin Concentration				
Time (Minutes)	Measured Color			
	Red	Green	Blue	Grayscale
1	0.67	0.96	0.49	0.82
10	0.37	0.13	0.06	0.30
